# Dexamethasone-mediated inhibition of Glioblastoma neurosphere dispersal in an ex vivo organotypic neural assay

**DOI:** 10.1371/journal.pone.0186483

**Published:** 2017-10-17

**Authors:** Ahmed M. Meleis, Aria Mahtabfar, Shabbar Danish, Ramsey A. Foty

**Affiliations:** 1 Department of Surgery, Rutgers Robert Wood Johnson Medical School, New Brunswick, New Jersey, United States of America; 2 Department of Neurological Surgery, Rutgers, New Jersey Medical School, Newark, New Jersey, United States of America; 3 Rutgers Cancer Institute of New Jersey, New Brunswick, New Jersey, United States of America; National Brain Research Centre, INDIA

## Abstract

Glioblastoma is highly aggressive. Early dispersal of the primary tumor renders localized therapy ineffective. Recurrence always occurs and leads to patient death. Prior studies have shown that dispersal of Glioblastoma can be significantly reduced by Dexamethasone (Dex), a drug currently used to control brain tumor related edema. However, due to high doses and significant side effects, treatment is tapered and discontinued as soon as edema has resolved. Prior analyses of the dispersal inhibitory effects of Dex were performed on tissue culture plastic, or polystyrene filters seeded with normal human astrocytes, conditions which inherently differ from the parenchymal architecture of neuronal tissue. The aim of this study was to utilize an ex-vivo model to examine Dex-mediated inhibition of tumor cell migration from low-passage, human Glioblastoma neurospheres on multiple substrates including mouse retina, and slices of mouse, pig, and human brain. We also determined the lowest possible Dex dose that can inhibit dispersal. Analysis by Two-Factor ANOVA shows that for GBM-2 and GBM-3, Dex treatment significantly reduces dispersal on all tissue types. However, the magnitude of the effect appears to be tissue-type specific. Moreover, there does not appear to be a difference in Dex-mediated inhibition of dispersal between mouse retina, mouse brain and human brain. To estimate the lowest possible dose at which Dex can inhibit dispersal, LogEC50 values were compared by Extra Sum-of-Squares F-test. We show that it is possible to achieve 50% reduction in dispersal with Dex doses ranging from 3.8 x10^-8^M to 8.0x10^-9^M for GBM-2, and 4.3x10^-8^M to 1.8x10^-9^M for GBM-3, on mouse retina and brain slices, respectively. These doses are 3-30-fold lower than those used to control edema. This study extends our previous in vitro data and identifies the mouse retina as a potential substrate for in vivo studies of GBM dispersal.

## Introduction

Despite advances in surgery, radiotherapy and adjuvant chemotherapy, glioblastoma remains an intractable disease with median survival of less than 30% at 1 year, 5% at 3 years and 3% at 5 years [1]. Most GBM patients relapse after undergoing the Stupp protocol [2], a widely used algorithm involving surgical resection of the primary tumor, followed by radiation therapy and Temozolomide (TMZ) chemotherapy. Unfortunately, aggressive and microscopic spread of tumor cells through the brain parenchyma renders GBM refractory to gross total surgical resection and targeted chemotherapy, leading to high rates of recurrence and overall poor prognosis and survival. Re-operation of recurrent disease yields weak added survival [3], due to the continued and ongoing spread of tumor cells, as well as increasing tumor cell resistance to TMZ chemotherapy. We believe that in order to help provide hope for possible increased survival and life expectancy, limiting the ability of GBM cells to disperse from the recurrence is paramount. A pharmacologic strategy using an agent that not only crosses the blood-brain-barrier with high bioavailability, but can also reduce dispersal of GBM cells would be ideal.

Previous publications provide compelling evidence that Dexamethasone (Dex), a drug that crosses the blood-brain-barrier and that is currently used to treat brain-tumor related edema in GBM patients, can also significantly reduce dispersal of immortalized [4] and primary [5] human GBM cells. Currently, the common standard of practice is to quickly taper and discontinue Dex treatment secondary to the adverse side effects of the medication [6]. While these findings are an important first step in testing the effects of Dex on GBM dispersal, the studies were conducted exclusively in vitro. Accordingly, the ECM was essentially kept constant, with variability perhaps arising as a consequence of cell-line specific secretion. Previous studies have shown that dispersal of cells from spheroids depends largely on a balance between forces that mediate cell-cell cohesion and cell-ECM adhesion [7], and that shifting this balance to favor cell-cell cohesion can significantly reduce detachment of cells from a primary mass [4, 5, 8]. Studies of ECM-specific migration in GBM have yielded paradoxical results. For example, studies testing single cell migration showed that laminin is they key modulator of GBM dispersal. [9, 10]. However, those studies utilized established high passage cell lines that had undergone some degree of clonal selection in vitro. Accordingly, many of the biological features of these high passage lines, including the capacity to interact with various ECM components, did not necessarily reflect the in vivo microenvironment [11]. Other studies using spheroids generated directly from several GBM biopsy specimens demonstrated that migration was highly variable between biopsy samples; in general, samples in which migration was strongly induced by laminin, were also stimulated by fibronectin, collagen type IV and vitronectin [12]. This suggests that migration is more a function of the biopsy specimen rather than of any particular component of the ECM.

Accordingly, the aim of this study was to utilize a reliable ex-vivo model to test 4 different neural substrates, extirpated murine retina, murine brain, porcine brain, and human brain slices, and their ability to stimulate migration and dispersal of cells from neurospheres. These assays offer tremendous value since they will provide insight on whether such models can be used as surrogate assays to explore the role of Dex, and possibly other drugs, on GBM dispersal prior to their use in vivo. By comparing dispersal on these substrates, we also wished to determine whether the mouse retina model would be an appropriate substrate for an in vivo assay in which human GBM cells are injected into mouse retina prior to Dex treatment. Finally, we wished to determine the lowest dose of Dex that reduces dispersal to understand and assess possible future dosing techniques in an in-vivo assay. Our aim is to bridge findings from our previous in vitro studies regarding the effect of Dex on GBM dispersal with a novel ex vivo model on various neural tissues that could, in principle, also translate in vivo.

## Methods

### Cell lines and drug treatment

The GBM-2 and GBM-3 cell lines used in this study were originally generated with approval of the Rutgers-Robert Wood Johnson Medical School Institutional Review Board under protocol #CINJ 001208. Samples were anonymized and the IRB waived the need for written informed consent. The lines used in this study have previously been published in [13]. GBM-2 and GBM-3 have been shown to be highly dispersive when placed as spheroids on tissue culture plastic. For standard dispersal assays, cells were either left untreated (UT) or were treated in 1x10^-7^M Dex for 24-hours prior to spheroid formation. For dose-response assays, GBM cells were treated for 24-hrs in 1x10^-6^M, 1x10^-7^M, 5x10^-8^M, or 1x10^-8^M Dex prior to spheroid formation. Where required, cells were incubated in 1x10^-7^M Dex or Dex and 1 μM RU-486 for 24 hours prior to spheroid formation.

### Generation of GBM neurospheres

Untreated and Dex-treated cells were detached from tissue culture plates at 80–90% confluence, washed several times in PBS, then stained with PKH-2 green fluorescent membrane intercalating dye. Spheroids were generated using the hanging drop method [14]. Cells were suspended at a concentration of 5x10^5^ cells/ml and 10 μl drops were deposited on the underside of a 60cm tissue culture dish lid. The lid was then inverted onto its base containing 5 mls of PBS for hydration. Hanging drops were incubated for 48–72 hours until spheroids formed.

### Isolation of mouse retinas

This study was carried out in strict accordance with the recommendations in the Guide for the Care and Use of Laboratory Animals of the National Institutes of Health. All work on mice was performed under an approved Rutgers IACUC protocol, #16–002. Retinas from C57BL mice (Charles River, Worcester, MA) were isolated as described in [15]. Briefly, mice were sacrificed by CO_2_ inhalation followed by cervical dislocation. Eyes were removed and fixed in 4% paraformaldehyde for 24 hours. Retinas were resected, washed in PBS then incubated in tissue culture medium for several hours prior to seeding with GBM spheroids.

### Generation of mouse, pig, and human brain slices

Brains from C57BL mice were dissected out immediately after euthanasia. The brain was removed en bloc with careful preservation. All work on pigs was performed under Rutgers IACUC protocol # 14–084, entitled “Surgical Techniques Training Lab”. These animals are used to train medical students and residents and are euthanized after each training session. Brains from teenage pigs were harvested immediately after animals were euthanised. Euthanasia was performed using an overdose of barbiturates in accordance with AVMA and Rutgers IACUC guidelines. Frontal lobes were removed en bloc, then cut into 1 cm sections. Mouse and pig brains were fixed in 4% paraformaldehyde for 24 hours, whereupon a Leica VT1000A vibratome was used to cut 100 micron thick slices. Slices were washed in PBS and incubated in tissue culture medium prior to seeding with GBM spheroids. Preserved frontal lobe from human brain measuring 1 cm^3^ were obtained from the Department of Neuroscience and Cell Biology, Rutgers-RWJMS, under an agreement with the New Jersey Anatomical Association. After reviewing, the Rutgers IRB determined that the study did not meet the regulatory definition of human subjects research provided in 45 CFR 46.102. Therefore, this project did not require formal approval by the IRB. Tissue blocks were sectioned into 100 micron slices, washed in PBS and incubated in tissue culture medium prior to seeding with GBM spheroids.

### Assessment of lateral migration of GBM spheroids

Fluorescently-labeled spheroids of GBM-2 or GBM-3 were deposited onto mouse retina, or onto mouse, pig, or human brain slices and incubated under tissue culture conditions for 24 hours (and 48-hours for human brain slices). Lateral migration distance was measured from images obtained by fluorescence microscopy. Using ImageJ, fluorescent signal was hand-traced. Total area of fluorescence for each spheroid was measured.

### Statistical analysis

**Fig 1:**
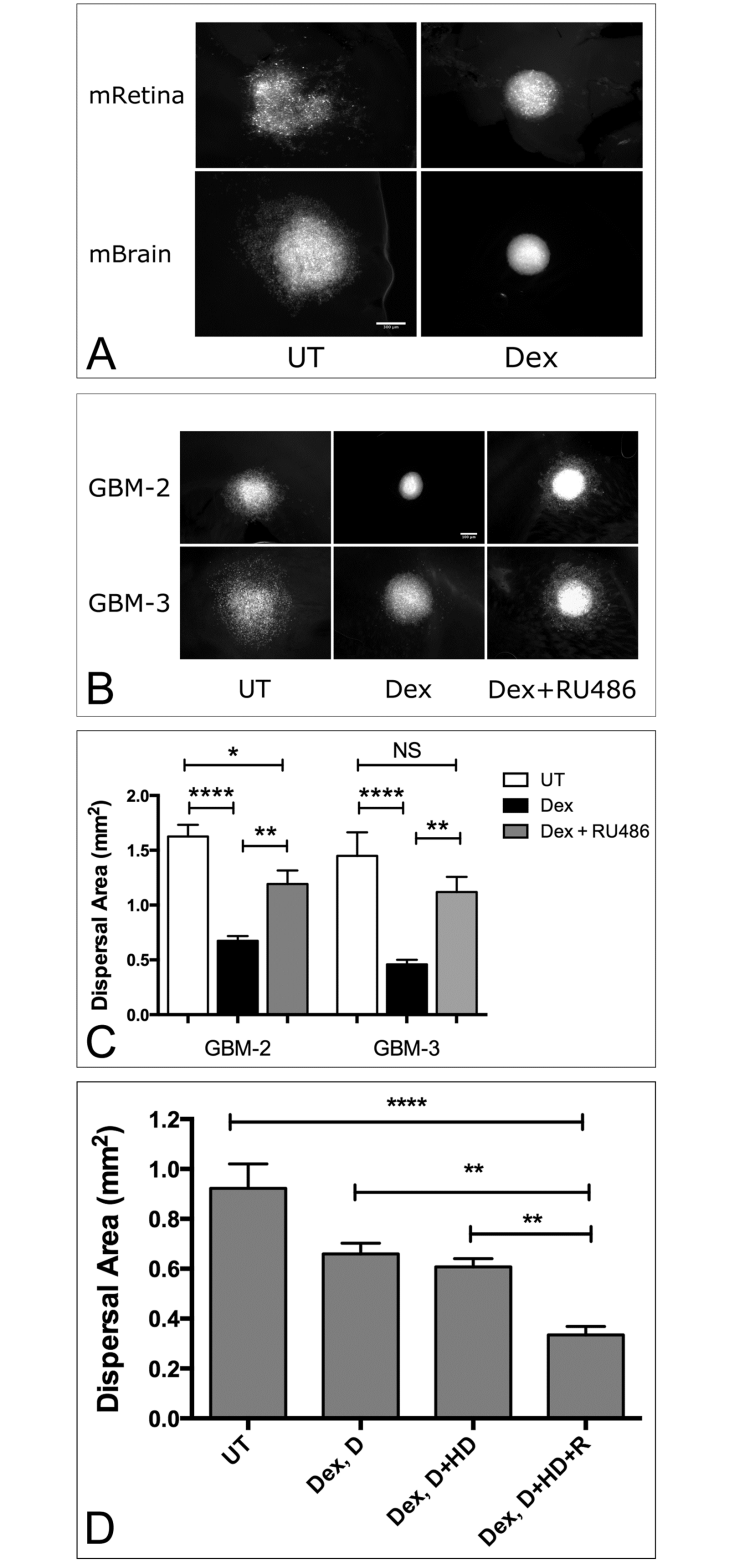
Dispersal of primary human GBM spheroids on extirpated mouse retina and brain slices. (A). Spheroids composed of PKH-2-stained cells measuring approximately 400 micrometers in diameter were deposited onto mouse retinas (mRetina), or 100 micron-thick, formalin-fixed mouse brain (mBrain) slices and incubated either in the absence or presence of 1x10^-7^ M Dexamethasone (Dex). After 24 hours in tissue culture, images were captured. Note significant dispersal of untreated (UT) spheroids relative to those treated with Dex. Scale bar = 300μm. Blocking the corticosteroid receptor reverses Dex-mediated inhibition of GBM dispersal on mouse brain (B). GBM-2 and GBM-3 spheroids were generated either in complete medium (UT), in the presence of 1x10^-7^M Dex, or Dex and 1μm of the steroid receptor antagonist, RU-486. Mean dispersal area was analyzed by ANOVA and Tukey’s HSD. For each data set, n = 12. Bars are standard error of the means. Asterisks represent significant difference of p<0.0001 (****) and p<0.001 (***). Dex withdrawal profile of GBM-3 on mouse retina (C). Dispersal area was measured for cells pre-treated in 2D culture with Dex (Dex, D) prior to spheroid formation, for cells pre-treated with Dex and as spheroids in hanging drop cultures (Dex, D+HD), or with Dex throughout the course of the experiment, including placement on mouse retina (Dex, D+HD+R). Bars represent standard error of the means. For each data set, n = 12. Average dispersal area was compared by One-Factor ANOVA, α of 0.05. Asterisks represent significant difference by Tukey’s HSD test (p<0.01 **).

**Fig 2:**
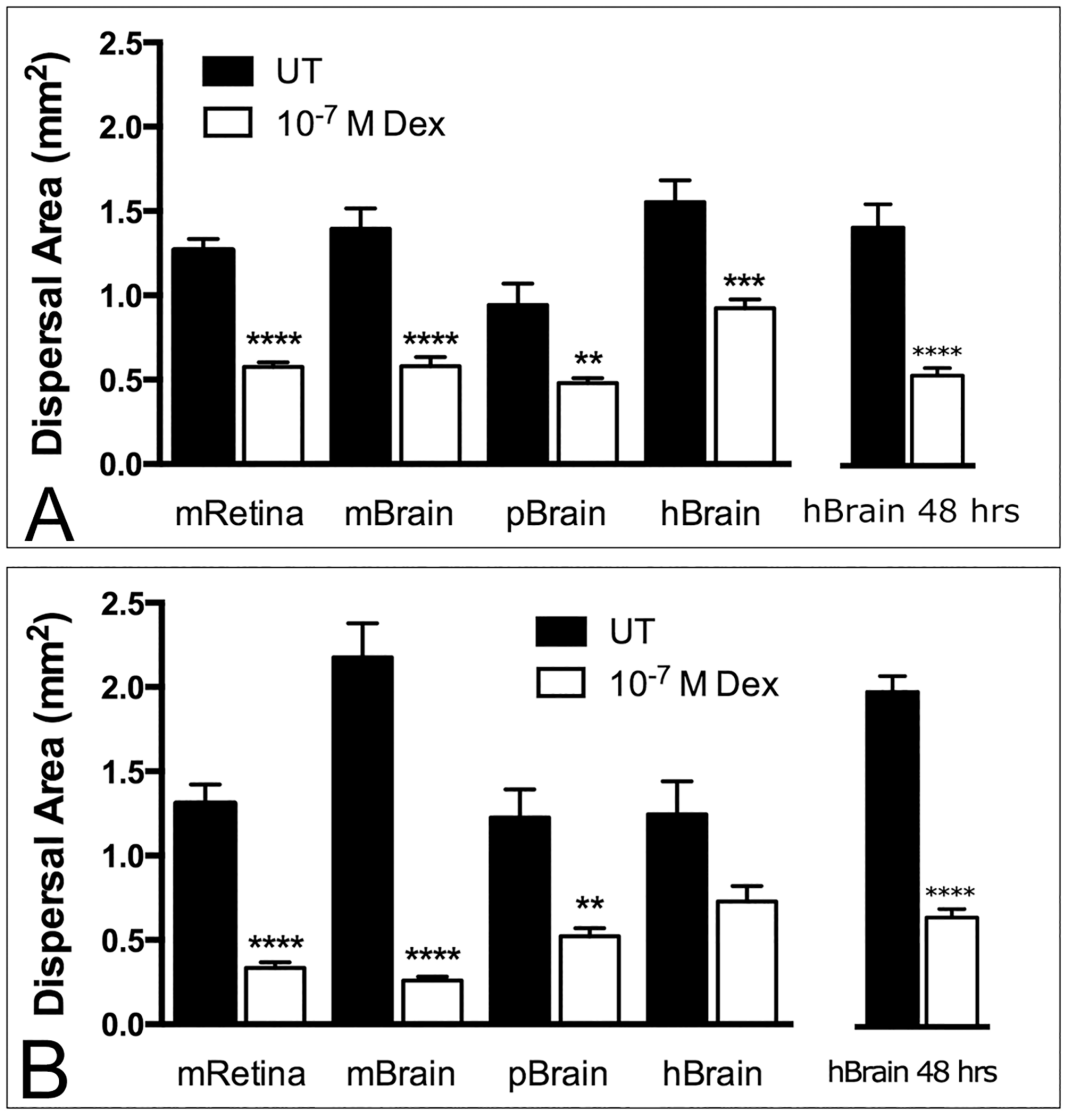
Dex-mediated inhibition of GBM dispersal on murine, porcine and human neural tissue. Fluorescently-labeled spheroids of GBM-2 (A) and GBM-3 (B) were placed onto extirpated mouse retina (mRetina), or onto 100 micrometer-thick slices of mouse Brain (mBrain), pig brain (pBrain), or human brain (hBrain) and incubated for 24 hours. Mean dispersal area was compared by Two-Factor ANOVA and Tukey’s HSD. Data sets ranged from n = 9 to n = 12, depending on outlier removal by ROUT Test. Asterisks represent significant difference in dispersal area between untreated and Dex-treated spheroids. Note that the effects of Dex on dispersal of GBM-3 on human brain did not reach statistical significance by Tukey’s HSD after 24 hours in culture, but did so after 48-hours (Fig 2B).

**Fig 3:**
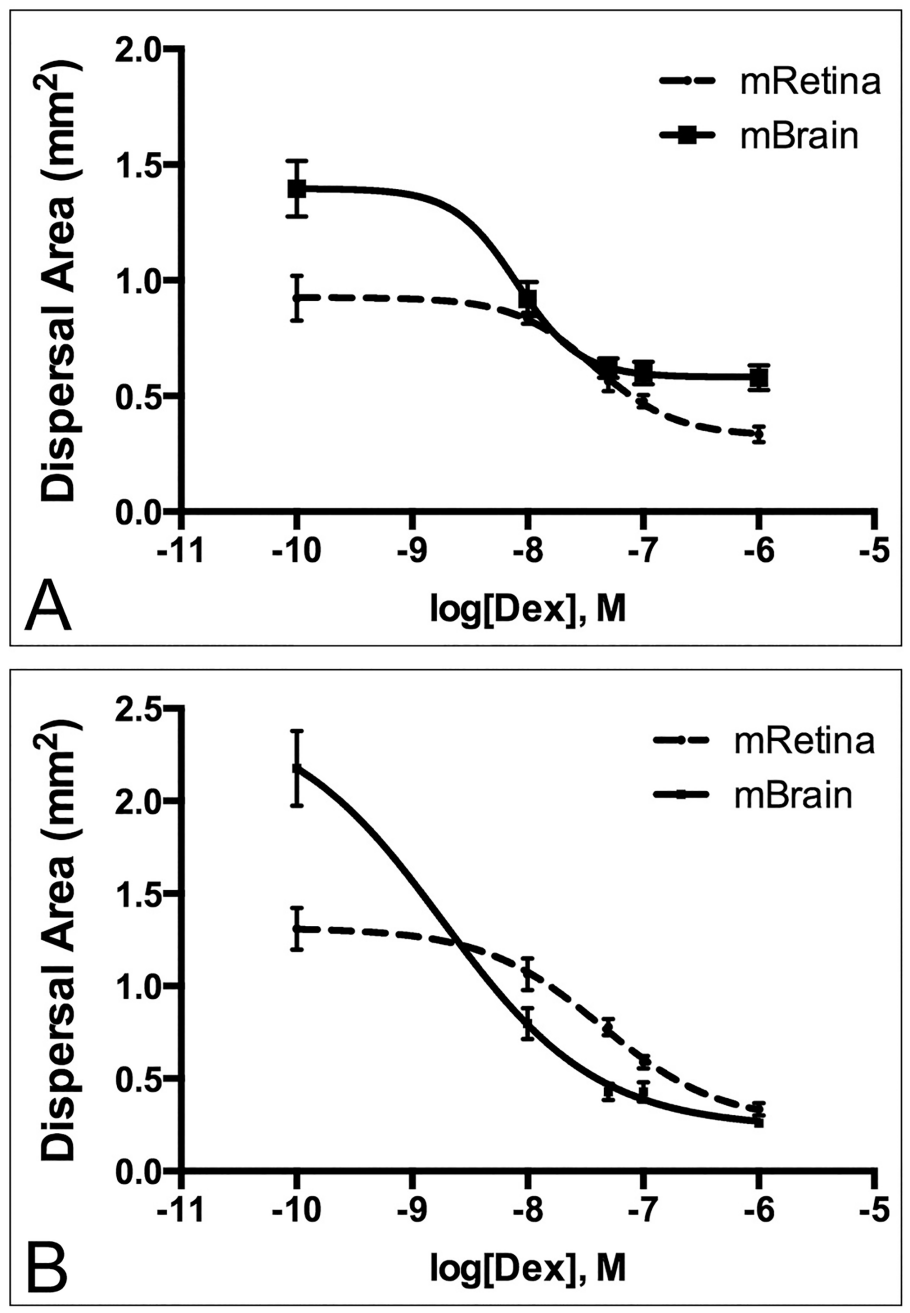
Dex dose-response curves of GBM dispersal on extirpated mouse retina and brain slices. Fluorescently-labeled spheroids of GBM-2 (A) and GBM-3 (B) were generated and plated onto mouse retina or brain slices in Dex concentrations ranging from 1x10^-10^ M to 1x10^-6^ M. Spheroids were incubated for 24 hours and dispersal area was plotted as a function of dose. For each data set, n = 10–12, depending on outlier removal by ROUT test. Curves were analyzed by non-linear regression. R-squared values for Goodness-of-fit ranged from 0.61 to 0.81. Log EC50 values were compared for mRetina and mBrain by Extra Sum-of-Squares F test (α of 0.05). p-values for differences in Log EC50 between mRetina and mBrain were 0.0033 and 0.0011 for GBM-2 and GBM-3, respectively.

One-Factor ANOVA was used to compare three or more group means (Fig 1C and 1D). Two-Factor ANOVA was used to test the effect of tissue type and Dex treatment on neurosphere dispersal (Table 1 and Fig 2). Tukey’s Honest Significant Difference (HSD) test was used to identify specific difference between means after ANOVA (Table 2). For dose-response curves between mRetina and mBrain (Fig 3) for GBM-2 and GBM-3, global non-linear regression was used to generate LogEC50 data, which were compared by the Extra Sum-of-Squares F test. For all tests, an α of 0.05 was used. Asterisks represent p<0.01 (**), 0.001 (***) and 0.0001 (****).

**Table 1:** p-values from a Two-Factor ANOVA for effect of tissue type and Dex-treatment on dispersal of GBM-2 and GBM-3. Analysis is based on n = 72 (9 neurospheres/tissue type, 4 tissue types, and 2 groups, untreated and Dex-treated), and 80 (10 neurospheres/tissue type, 4 tissue types, and 2 groups, untreated and Dex-treated) for GBM-2 and GBM-3, respectively, and an α of 0.05.

	Tissue Type	Dex Treatment
GBM-2	p<0.0001	p<0.0001
GBM-3	P<0.0069	P<0.0001

**Table 2:** Results of Tukey’s MCT following One-Factor ANOVA for comparison of dispersal of untreated (UT) and Dex-treated (Dex) GBM-2 and GBM-3 on mouse retina (mR), mouse brain (mB) and human brain (hB) slices. Analysis is based on n = 98 GBM-2 and 107 GBM-3 neurospheres (8–12 neurospheres/tissue type, 4 tissue types, and 2 groups, untreated and Dex treated). NS = not significant at α of 0.05.

	UTmR-UTmB	DexmR-DexmB	UTmR-UThB	DexmR-DexhB
GBM-2	NS	NS	NS	NS
GBM-3	P<0.0001	NS	NS	NS

## Results

### An ex vivo model of GBM dispersal on various neural substrates

We have previously shown that spheroids of GBM cells placed on tissue culture plastic can readily disperse and that Dex significantly reduces dispersal [4, 5]. These earlier studies were conducted exclusively on tissue culture plastic. To demonstrate potential translation to in vivo models, it is important to first develop ex vivo models that are able to rigorously quantify microscopic dispersal of GBM cells using relevant biological tissues. In this study, we tested whether spheroids composed of human primary GBM cells were able to disperse upon various tissue substrates, including mouse retina and brain slices of murine, porcine and human origin.

As proof of principle, we first show that aggregates of GBM readily spread on formalin-fixed mouse retina and brain slices and that the pattern of dispersal differs in response to tissue type. GBM cells appear to disperse as chords of cells on mouse retina, but as advancing sheets with some single cell detachment on mouse brain. Dex-treated aggregates, however, remain localized and cells fail to disperse to any significant extent past the spheroid boundary (Fig 1A).

To further test the model, we asked whether dispersal inhibition was Dex-specific. Since Dex works through interaction with corticosteroid receptors, we used the corticosteroid receptor antagonist, RU-486, to determine whether blocking the interaction between Dex and its’ receptor would effectively “rescue” dispersal. We first performed a dispersal assay of GBM-3 on mouse brain to test whether DMSO, the solvent for RU-486, or RU-486 influence dispersal. Neither DMSO or RU-486 inhibit dispersal (ANOVA, p = 0.3006, S1 Fig). We then compared mean dispersal area for GBM-2 and GBM-3 on slices of mouse brain for untreated neurospheres or those treated with 1x10^-7^M Dex (Dex) or a combination of Dex and 1 μm RU-486 (Dex+RU-486). Neurospheres treated with the combination of drugs appear to spread in a manner similar to untreated neurospheres, suggesting a “rescue” of dispersal (Fig 1B). To quantify this apparent “rescue” of dispersal, one-factor ANOVA and Tukey’s HSD were used to compare mean dispersal area between groups. For GBM-3, Dex treatment, as expected, gave rise to a significant decrease in dispersal area. However, in the presence of Dex and RU-486, no significant difference was detected between untreated neurospheres and those treated in a combination of Dex and RU-486. For GBM-2, combination treatment also resulted in a marked increase of neurosphere dispersal area, but not to levels demonstrated by untreated samples (Fig 1B). Of note, is that mean dispersal area of Dex-treated GBM-2 neurospheres was significantly smaller than for those treated with Dex and RU-486. This strongly suggests that dispersal was effectively rescued, but perhaps not to levels comparable to the dispersal exhibited by untreated neurospheres. That RU-486 was able to effectively “rescue” dispersal on fixed tissues indicates that the effects of substrate dispersal are Dex-specific. To establish whether Dex must be continuously available to cells in order to inhibit dispersal, we generated a Dex withdrawal profile for GBM-3 on mouse retina. Here, cells were incubated in Dex throughout the course of the experiment or it was excluded at different time points in the assay. We then assessed dispersal area as a function of how long Dex remained in the medium over the time course of the experiment: in tissue culture dish (Dex, D), in tissue culture dish and hanging drop (Dex, D +HD), or in tissue culture dish, hanging drop, and on retina (Dex, D+HD+R). Maximal effect on inhibition of dispersal was observed when Dex was included throughout the assay (Dex, D+HD+R). Effect on dispersal inhibition was less pronounced if Dex was excluded at any time point in the assay (ANOVA, Tukey’s Honestly Significant Difference (HSD) test, p<0.05, Fig 1D).

### Dexamethasone inhibits GBM dispersal on mouse and human neural tissue

Previous studies demonstrated that Dex-treatment significantly reduced dispersal of GBM spheroids deposited on tissue culture plastic. Here, we explored whether Dex would have a similar effect when GBM spheroids were deposited on mouse, pig, and human neural tissue. Dispersal of GBM-2 and GBM-3 on mouse retina, or on 100 micron-thick slices of mouse, pig, or human brain was measured after 24 hours in culture. A two-way ANOVA tested the effects of Dex-treatment and type of neural tissue on spheroid dispersal. The effect of both Dex treatment and tissue type on dispersal of GBM-2 and GBM-3 were found to be significant (p<0.0001, Table 1). Tukey’s HSD test was then used to identify specific differences in mean dispersal between untreated and Dex-treated spheroids on the four tissue types. For both GBM-2 (Fig 2A) and GBM-3 (Fig 2B), Dex-treatment significantly reduced dispersal on mouse retina and slices of mouse brain and pig brain. Whereas Dex also appeared to reduce dispersal of both lines on human brain, the difference was not statistically significant by Tukey’s HSD for GBM-3. However, when analysed by Fisher’s LSD or by pairwise t-test, significance was detected after 24 hours. This statistical discrepancy was likely due to slower spreading of GBM-3 on human brain slices. Indeed, when experiments on human brain slices were allowed to proceed for 48 hours, statistical significance was detected after One-Factor ANOVA and Tukey’s HSD test for both GBM-2 (Fig 2A) and GBM-3 (Fig 2B).

### Mouse retina is a good substrate for GBM dispersal

In order to determine whether the mouse retina model is a good alternative to brain tissue, we measured and compared dispersal of untreated and Dex-treated GBM-2 and GBM-3 spheroids on mouse retina, mouse brain, and human brain. Analysis of the dispersal data shows that spheroids of untreated GBM-2 spread equally well on all substrates, and that Dex was equally effective in reducing the amount of spread on each substrate. Similar results were observed for GBM-3, with the exception that spheroids appeared to spread more readily on mouse brain than on mouse retina (Tukey’s HSD, p = 0.0011, Table 2), perhaps because GBM-3 is able to adhere more quickly to mouse brain tissue.

### Dose-dependent dispersal of GBM in mouse retina and brain slice assays

In previous assays, we used a single Dex dose to explore effects on dispersal. We chose 1x10^-7^M, a dose that corresponds to a 4mg/day oral dose. In this study, we wished to determine the lowest possible dose that could still inhibit dispersal. We bracketed the dosage to include concentrations as high as 1x10^-6^ M and as low as 1x10^-10^M (an effective 0 dose). We tested both GBM-2 and GBM-3 cells but only plated aggregates on mouse retina and mouse brain slices as these tissues could ultimately be used as injection sites for in vivo mouse assays. Dispersal area was plotted as a function of dosage and the curves analyzed by non-linear regression. LogEC_50_ values were compared by Extra Sum-of Squares F-test. For GBM-2, the EC50 Dex dose on mouse retina and brain slices differed significantly (p<0.0033). Fifty percent dispersal of GBM-2 was achieved with doses of 3.79x10^-8^M and 8.04x10^-9^M, respectively (Fig 3A). Similar results were obtained for GBM-3, with EC50 values for retina and brain of 4.3x10^-8^M and 3.4x10^-9^M, differing significantly (p<0.0011. Fig 3B).

## Discussion

Glioblastoma (GBM) is a proliferative, angiogenic, necrotic and importantly, a dispersive disease. GBM cells disperse to distal sites throughout the entire brain by invading the CNS parenchyma individually or as groups of cells forming a network throughout the neuropil [16]. Correlation of histological sections of human brains with radiologic images has shown that tumor cells can present several centimeters outside the enhancing area on MRI [17]. Therefore, in order move towards longer survival time for GBM patients, limiting the ability of GBM cells to disperse is of paramount importance. A pharmacologic strategy using an agent that can cross the blood-brain-barrier, has high bioavailability, and can reduce dispersal of GBM cells would be ideal. Previous in- vitro results have shown that Dexamethasone can significantly inhibit dispersal of GBM cells. Whether Dex can elicit a similar response in vivo is currently unknown. To address this question requires development of an appropriate animal model that can mimic the cellular and ECM microenvironment that GBM cells encounter as they disperse.

Standard xenograft assays in which tumor cells are injected or implanted into the flanks of immune-compromised mice are inadequate since the site of injection does not mimic the cellular architecture in which GBM cells migrate. Performing xenograft assays in which GBM cells are injected into mouse brains is not ideal since visualizing the tumor cells for analysis of dispersal would require removal of the brain and histological preparation requiring hundreds if not thousands of histologic serial sections in order to detect dispersing cells. In vivo animal imaging systems cannot be used since they are generally only useful for measuring gross tumor size and lack the resolution to detect cells that disperse either in small groups or as single cells. For these reasons, an assay that would allow high-resolution confocal microscopy to specifically quantify the lateral migration distance in response to Dex treatment is ideal. However, such as assay would require a tissue substrate that could be easily adapted to analysis by epi-fluorescence or confocal microscopy. A mouse retina model is ideal and offers several advantages: 1) retina is neural tissue [18], so GBM cells will be in an environment that approximates the physical microenvironment into which they disperse; 2) The overall retinal thickness in a C57BL/6 mouse model was determined to be 204.41 ± 5.19 μm [19], making extirpated retina amenable to confocal or two-photon microscopy. This would allow for quantification of both lateral migration and z-axis penetration distance of fluorescently-labeled GBM cells in response to drug treatment; 3) Our data show that human GBM cells readily spread on mouse retina and that the process takes place in as little as 24 hours. This is significantly less time than for typical xenograft models; 4) Since the eye is an immune privilege site [20], studies can be performed on fully immune competent animals without risk of rejection; 5) Injected retinas can be recovered and assessed for fibronectin matrix assembly and other markers. This will help establish the Dex-mediated effects at the molecular level in an vivo system. However, prior to developing the in vivo assay, it was important to first establish proof-of-concept in an ex vivo system. Of paramount importance was determining whether retina is a good substitute for brain tissue.

We first determined whether GBM spheroids spread onto extirpated mouse retina and mouse brain slices. Spheroids dispersed equally well on both. This suggests that it may be possible to use retina as a substrate for GBM dispersal. Interestingly, GBM cells appear to disperse as chords of cells on mouse retina, but as advancing sheets with some single cell detachment on mouse brain. This argues that the retina model may be superior to brain when conducting single cell dispersal assays. Our observation that Dex treatment inhibits dispersal of GBM on both substrates confirms utility of the retina model as a potential drug-testing platform. That RU-486 was able to effectively “rescue” aggregate dispersal on fixed retina and brain tissue indicates that the effects of substrate dispersal are Dex-specific. However, because RU-486 is an antagonist of both glucocorticoid and progesterone receptors [21], its’ action in the presence of Dex, does not rule out the possibility that the “rescue” of dispersal might be happening through a glucocorticoid-independent mechanism.

Clinically, patients are administered Dex QID to maintain serum titers [6]. Accordingly, we asked whether Dex needs to be continuously available in the medium in order to inhibit dispersal of GBM cells. This was necessary in order to assess whether the cell biology effect on the tumor cells could just be activated with a one-time exposure to Dex or if continued exposure to Dex was necessary. We show that removing Dex at any time point in the experiment resulted in an attenuated effect on dispersal. This suggests that with future in vivo assays, it will be important to carefully monitor the timing of Dex administration to help maintain sufficient titers for its’ maximal dispersal inhibitory effect.

As mentioned in the introduction, the ECM is an important regulator of GBM dispersal [9, 10, 12]. Typically, studies exploring the role of the ECM in mediating dispersal rely on interaction between tumor cells and purified ECM-coated tissue culture plastic. However, in vivo, the substrate over which GBM cells must migrate is much more complex. Accordingly, we chose to 4 neural tissue substrates to identify potential preference of GBM cells to substrate. We chose mouse retina, mouse brain, porcine brain, and human brain. We wished to first determine if untreated GBM cells displayed any difference in dispersal on the 4-substrates. A two-way ANOVA tested the effects of Dex-treatment and type of neural tissue on spheroid dispersal. The effect of both Dex treatment and tissue type on dispersal of GBM-2 and GBM-3 were found to be extremely significant (p<0.0001), as was the interaction between those two variables (p<0.0020 and <0.0001 for GBM-2 and GBM-3, respectively, Table 1). Tukey’s HSD test was then used to identify specific differences in mean dispersal between untreated and Dex-treated spheroids on the four tissue types. For the most part, GBM cells appear to disperse equally well on all 4 substrates, although for GBM-3, dispersal appears to be better on mBrain than on other substrates. Moreover, the effects of Dex appear to be similar across the 4 substrates. Although, while Dex appeared to reduce dispersal of GBM-3 on human brain, the difference was not statistically significant by Tukey’s HSD if dispersal was assessed after 24 hours. However, when dispersal was allowed to proceed for 48 hours, statistical significance was detected by Tukey’s HSD. This discrepancy could be possibly explained by higher overall cohesion of GBM-3 aggregates [5]. Higher aggregate cohesion could effectively restrain cell dispersal from the mass and reduce the overall extent of dispersal over time. Alternatively, spheroids of GBM-3 may require more time to attach to the brain ECM. This could also influence extent of dispersal over time. Collectively, these results suggest that GBM migration can take place on different neural tissue derived from 3 different species, and that Dex can inhibit dispersal independently of substrate. These results suggest that it may be possible to translate the *ex vivo* mouse retina data, to an *in vivo* mouse retina model.

A pharmacologic approach using Dex is logical inasmuch as it is an already approved palliative therapy for brain tumors. Despite its efficacy in reducing edema, side-effects of Dex treatment often limit its long-term use, particularly at doses typically used to reduce edema. Accordingly, patients are typically weaned off the drug once edema has resolved [6]. Recent studies have also suggested that the anti-proliferative effects of Dex may shorten survival in Glioblastoma by conferring protection from radio-therapy and chemotherapy- induced genotoxic stress [22]. Thus, in order to optimize the inhibitory effects of Dex on dispersal, while also reducing potential risk of interfering with other treatment modalities, we interrogated the lowest possible Dex dose that could inhibit dispersal of GBM in our ex vivo model. We therefore generated dose response curves in order to assess the relationship between Dex dose and dispersal on two of the four tissue substrates. We chose mRetina and mBrain since these could likely be the next step in developing an in vivo model. A typical therapeutic oral dose of Dex to reduce edema is between 4 mg and 16 mg/day [6]. The predicted concentration of Dex in the brain 6 hours after a 0.5 mg oral dose of Dex peaks at 5x10^-3^ μg/ml [23]. This is equivalent to a 1.3x10^-8^ M in vitro concentration. Therefore, a 4-mg dose is equivalent to an in vitro concentration of 1x10^-7^ M. We bracketed the dosage to include concentrations as high as 1x10^-6^ M and as low as 1x10^-10^M. EC50 values demonstrate that on mouse retina, it is possible to achieve 50% reduction in dispersal with doses ranging from 2.3 to 2.6-fold lower than the 4mg/day dose. For mouse brain, much lower doses are needed to achieve a 50% reduction in dispersal, with doses ranging from 12.4 to 29-fold lower for GBM-2 and GBM-3, respectively. Therefore, it seems that more Dex is needed to maintain the tumor cell-cell cohesion needed to inhibit dispersal and overcome adhesions and dispersal of tumor cells on the retina ECM. This means that if Dex can stop the dispersal effect of tumor cells on retina, it will likely be just as effective, if not more effective, in brain. However, one must consider that possible differences in bioavailability of Dex between mouse retina and brain in an in-vivo scenario. Nevertheless, the values established by the ex vivo model provide a good starting point to establish dose ranges for future in-vivo experiments.

## Conclusions

The goal of any ex-vivo or in vivo animal model, is to eventually be translated into a clinical trial. Accordingly, we also compared efficacy of Dex in reducing dispersal of GBM on human brain slices. Importantly, our ex-vivo assay showed that there was no statistical significance in dispersal of untreated and Dex treated GBM-2 and GBM-3 on mouse retina and human brain. GBM aggregates were able to adhere to the neural substrate of retina and brain and spread with equal efficacy. Moreover, Dex was able to inhibit dispersal of tumor cells on both substrates with equal efficacy. This suggests that the mouse retina is a good neural substrate for GBM dispersal and sufficiently mimics human brain parenchyma.

## Supporting information

S1 Fig DMSO and RU-486 do not inhibit dispersal of GBM-3 neurospheres on mouse brain slices. Fluorescently-labeled neurosphers of GBM-3 were generated either in complete medium (UT, n = 9), in DMSO (the carrier for RU-486, n = 10) or in 1 μm RU-486 in DMSO (n = 8), deposited onto mouse brain slices, and incubated for 24 hours whereupon dispersal area was measure as previously described. Mean dispersal area for each group was analyzed by ANOVA. No difference in mean dispersal area was detected (p = 0.3006).
(TIFF)
