# Supplementary figures and images for: Dexamethasone-mediated inhibition of Glioblastoma neurosphere dispersal in an ex vivo organotypic neural assay
Source: PLoS One. 2017 Oct 17;12(10):e0186483. doi: 10.1371/journal.pone.0186483 (PMC5645119; doi:10.1371/journal.pone.0186483)

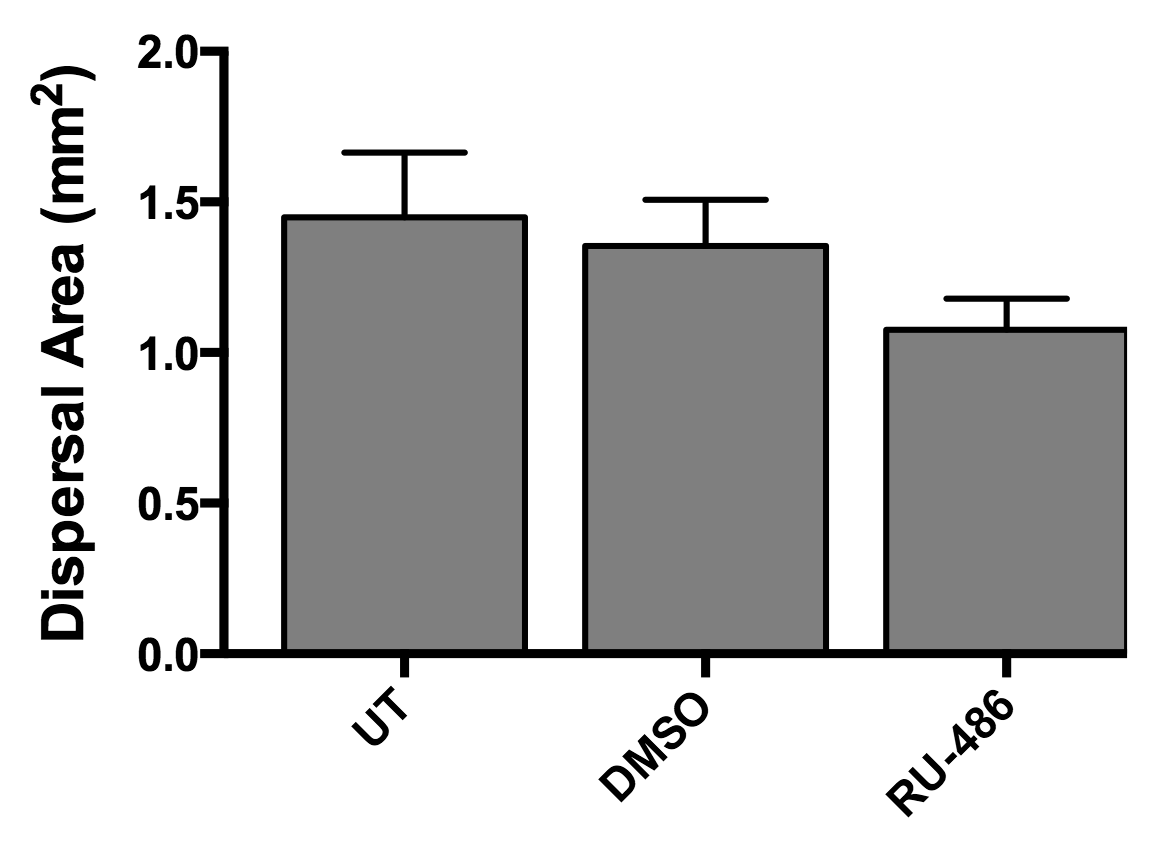

Supplement: S1 Fig — Fluorescently-labeled neurosphers of GBM-3 were generated either in complete medium (UT, n = 9), in DMSO (the carrier for RU-486, n = 10) or in 1 μm RU-486 in DMSO (n = 8), deposited onto mouse brain slices, and incubated for 24 hours whereupon dispersal area was measure as previously described. Mean dispersal area for each group was analyzed by ANOVA. No difference in mean dispersal area was detected (p = 0.3006). (TIFF) [file pone.0186483.s001.tiff]
